# Comprehensive Review on Mechanical Performance of Concrete Reinforced with Fibers and Waste Materials

**DOI:** 10.3390/ma18235419

**Published:** 2025-12-01

**Authors:** Rajesh Kumar Mishra, Hafsa Jamshaid, Miroslav Muller, Jiri Urban, Michal Penc

**Affiliations:** 1 Department of Material Science and Manufacturing Technology, Faculty of Engineering, Czech University of Life Sciences Prague, Kamycka 129, Suchdol, 165 00 Prague, Czech Republic; muller@tf.czu.cz (M.M.); urbanjiri@tf.czu.cz (J.U.); penc@tf.czu.cz (M.P.); 2 Department of Textiles, School of Engineering & Technology, National Textile University, Faisalabad 37610, Pakistan

**Keywords:** construction materials, global warming, high elastic modulus fiber, low elastic modulus fiber, fibrous waste, biodegradability, non-biodegradable, mechanical properties

## Abstract

Concrete is one of the most important and most widely used materials for construction activities around the world. However, it has inherent deficiencies, e.g., brittleness, low impact resistance, low tensile strength, low fire resistance, low durability, and lower resistance to crack formation. Fibers and waste materials of different types are added as partial replacement of cement and aggregates in concrete to improve performance properties and reduce environmental pollution. In the present study, a thorough review of the use of various types of fibers with high and low elastic moduli in concrete to improve mechanical performance and reduce environmental pollution issues has been conducted. This review paper also provides comprehensive information on the different types of waste materials, e.g., biodegradable and non-biodegradable, which are used in concrete. The use of waste materials in concrete reduces the amount of waste sent to landfill and, in addition, improves some mechanical properties of concrete. This review is aimed at evaluating and understanding the strengths and weaknesses of fiber-reinforced concrete by using SWOT (Strengths, Weaknesses, Opportunities, Threats) analysis. Moreover, this study also concluded that carbon fiber-reinforced concrete proves to be stronger and more durable but more expensive than other fibers. An ideal percentage of natural origin fibers used in concrete can greatly improve the mechanical performance. This study also discussed that waste from polymeric materials can be used in concrete as a partial replacement of cement and other components, e.g., coarse aggregates. It can be inferred that the optimum content of fibers that gives effective results is about 1%, and the reinforcement of concrete with different varieties of wastes as a replacement for fine aggregates should not be more than 2%. Parametric optimization of fiber content will be necessary for the best possible combination of performance properties.

## 1. Introduction

Cement and concrete materials are widely used for construction works, surface finishing, and facade elements. It consists of water, sand, cement, and coarse aggregates. It can be considered as the second most widely required material globally after food [1]. As per the World Business Council report for sustainability, more than 3.8 tons of cement and concrete are used annually per person worldwide [2]. The increasing use of concrete has serious consequences for the environment. Production of cement emits an enormous amount of CO_2_, which is a major part of the total CO_2_ emissions globally [3].

Concrete shows a brittle behavior with relatively poor tensile strength, which can lead to sudden tensile failure, which is obviously not desirable for any construction material used in structural applications. There are several approaches to overcome this issue, one of which is to replace some components in the concrete with other reinforcement materials, but concrete has undisputable advantages, and complete replacement is not possible. Partial replacement of concrete components with alternative materials that are eco-friendly is a viable solution to this global problem. Historically, steel has been used for the reinforcement of concrete. Steel reinforcement used in concrete also affects the environment through energy consumption and emission of greenhouse gases during its processing. In addition, steel is prone to corrosion and has a high weight; that is why engineers and researchers are focusing on providing economical, light-weight, environment-friendly, and non-corrosive concrete materials. Textile and other waste fibers can be used in concrete, and they prove to be a better option for partial replacement of concrete components, e.g., fine and coarse aggregates [4]. Fiber-reinforced concrete materials are one kind of composite material consisting of normal concrete reinforced by random, short, discontinuous fibers. Fibers can partially replace some of the components in concrete, e.g., cement, steel, and coarse aggregates. Fiber-reinforced concrete is lighter in weight and may have better mechanical (tensile, compression, flexural, ductility) and chemical properties (resistance to corrosion, chemical resistance) compared to normal concrete. The tensile strength, flexural strength, and compressive strength of concrete can be improved when fiber-based reinforcement is added [3,4]. The use of fiber helps to reduce crack growth/width in the construction. The addition of fibers to concrete can also improve the ductility, along with improved post-cracking load-carrying capacity.

In the present era, researchers are focusing on the issue of sustainability in construction-related materials. The definition of sustainability is to satisfy the current needs without compromising the rights of future generations. It also refers to the capacity for human civilization and Earth’s biosphere to co-exist. According to the Sustainable Development Goal (SDG), the agenda for 2030 is to change the world for the better. The sustainability of construction materials needs a blend of new and old materials [5].

The use of renewable and recyclable waste materials in construction works to reduce the cost of the material and handle solid waste disposal is a multidirectional approach. The waste materials produced by industries need to be used in an environmentally friendly way. Some of the industrial wastes can be utilized in construction works as a partial replacement to manage the waste-handling problems. Use of waste materials in concrete not only solves the waste-handling problem but also imparts positive performance benefits in construction. Some types of waste materials, when used in concrete, can improve ductility behavior, reduce overall weight, and partially improve the mechanical performance of concrete. Tons of waste are produced by textile and other industries, which causes serious environmental issues. According to the global textile waste statistics, almost 110 million tons of textile waste are produced each year [6,7]. Textile wastes used in concrete reduce the weight and enable better performance of the construction material.

About 1.3–1.5 billion tons of rubber tire waste is being produced every year, and this figure is increasing day by day [8]. Waste tire material causes serious environmental issues when burned in the open air or disposed of as landfill. The use of tire waste in the construction industry is a perfect solution to reduce the handling of such waste while decreasing the brittleness of the concrete. Waste of plastic materials increased abruptly during the COVID-19 pandemic, which was estimated at 1.6 billion tons per day [9]. The PET (polyethylene terephthalate) bottle waste and facial mask waste may be utilized in the construction industry as a partial replacement of concrete components. Plastic waste used in concrete may improve compressive and tensile strengths. The aim of this review is to provide comprehensive information on the use of different types of fibers and waste materials in concrete with a view to reducing the cost of the concrete material and effectively utilizing different waste materials in an economically and ecologically beneficial way. The influence of fiber reinforcement and various types of waste materials on the cracking mechanisms, compressive, flexural, and tensile strengths, slump value, resistance to chemical environment, durability, and cost of the concrete is also analyzed. The possible applications of concrete containing fibers or waste materials are discussed, and suggestions are given about the best option for their potential applications [10].

## 2. Methodology

**Figure 1:**
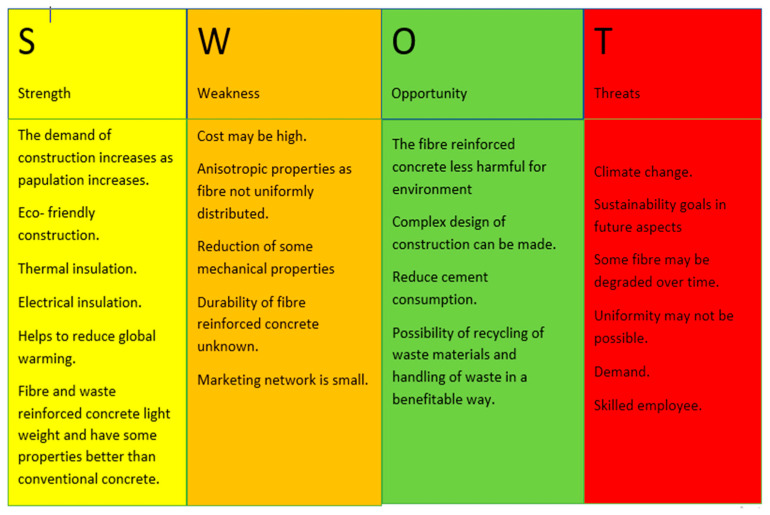
SWOT analysis of fiber-reinforced concrete.

In this review paper, relevant articles related to the use of fibers and waste materials as reinforcement in concrete were collected from various trusted sources. The fundamental field of interest was represented and identified by different keywords. The keywords of search were “waste reinforced concrete”, “mechanical properties of concrete using natural fibre”, “natural fibre reinforced concrete”, “natural and synthetic fibres in concrete”, “synthetic fibre reinforced concrete”, “polyester reinforced concrete”, “industrial waste reinforced concrete”, “textile waste reinforced concrete”, “waste tire rubber in concrete”, “use of waste tire rubber in concrete”, etc. The related literature and articles were gathered and analyzed in detail based on the search keywords. The necessary and related data about the chosen topic was collected from the literature and classified in a systematic manner. SWOT (Strengths, Weaknesses, Opportunities, Threats) analysis of the collected information was performed for analysis of the usage of the fiber-reinforced concrete and waste material-based reinforcement material for concrete and construction in the future to fulfill the Sustainable Development Goals (SDGs). SWOT analysis is one of the best tools for economic and strategic management, as shown in Figure 1.

## 3. Fibers Used in Concrete

Fibers can be used as reinforcement in concrete to delay the formation of cracks and to reduce the brittleness of concrete structures. Fibers can also partially replace some of the concrete components and reduce cement consumption, which greatly reduces the CO_2_ emission during the whole process [7,10]. Fibers can be categorized in different ways, e.g., according to their modulus of elasticity or their origin. Based on the modulus of elasticity, the classification can be referred to as Higher elastic modulus (HEM) and Lower elastic modulus (LEM). Higher elastic modulus (HEM) fibers, like steel, carbon, and glass, are those whose modulus is higher than that of the concrete mix, while Lower elastic modulus fibers (LEM), like polypropylene, polyester, polyamide, polyethylene, and vegetable fibers, have a lower modulus than that of the concrete mix. Different fibers can be used as reinforcement in concrete, which can enhance the physical properties of the construction material.

### 3.1. High Elastic Modulus (HEM) Fibers

High-performance fibers or high elastic modulus fibers are those that have some unique properties or a higher level of mechanical properties compared to concrete. Typically, a high-performance fiber shows relatively high tensile strength, temperature resistance, chemical resistance, and abrasion resistance. Some examples of such high-performance fibers are carbon, para-aramid, basalt, and glass fibers. These fibers are used in aerospace engineering, textile engineering, construction engineering, protective clothing, and civil engineering, among others. HEM can simultaneously improve the flexural and impact resistance of concrete structures, while low elastic modulus (LEM) fibers can improve the impact properties only. Some of the details of HEM and LEM fibers are discussed in ongoing sections. Mechanical properties of some selected Higher elastic modulus (HEM) fibers are listed in Table 1.

#### 3.1.1. Glass Fiber

Glass fiber is lightweight, ductile, and exhibits high thermal and fire resistance. Applications of glass fiber are in automotives, aerospace, chemical, and construction industries. Glass fiber has superior tensile strength and proves to be better when used in concrete. Concrete with glass fiber reinforcement can be lighter, have higher compressive strength, lower crack formation, and show a higher strength-to-weight ratio. Several researchers have used glass fiber as reinforcement and conducted tests for mechanical strength. It has been concluded that the compressive and flexural strengths of glass-reinforced concrete were increased when the fiber percentage increased to 1.5%. Compressive strength of concrete can be reduced by 20% when the glass fiber percentage is below or above 1.5% [13]. Increasing the percentage of glass fiber can increase the number of defects in concrete, which may result in inferior mechanical properties. Therefore, the optimum amount of glass fiber used in concrete that can improve the required mechanical performance is 1.5%. Durability of concrete is increased by the addition of glass fibers, although it is slightly more expensive than ordinary concrete. Compressive strength of glass fiber-reinforced concrete increased by 20 to 25% after 22 days of curing of the samples. There was up to a 20% increase in bending strength after 22 days of curing of the concrete samples. The bleeding of glass fiber-reinforced concrete is reduced, which, in turn, improves the surface integrity of concrete. Toughness and resistance to crack formation inside the concrete were improved by reinforcement of glass fiber in concrete [14]. The tensile strength increases when glass fibers are used in concrete, and, thus, makes it tougher and more ductile compared with normal concrete [15].

Inclusion of glass fiber in concrete can improve durability. Glass fiber-reinforced concrete (GFRC) can be used in exterior structures of buildings [16].

#### 3.1.2. Basalt Fiber

**Figure 2:**
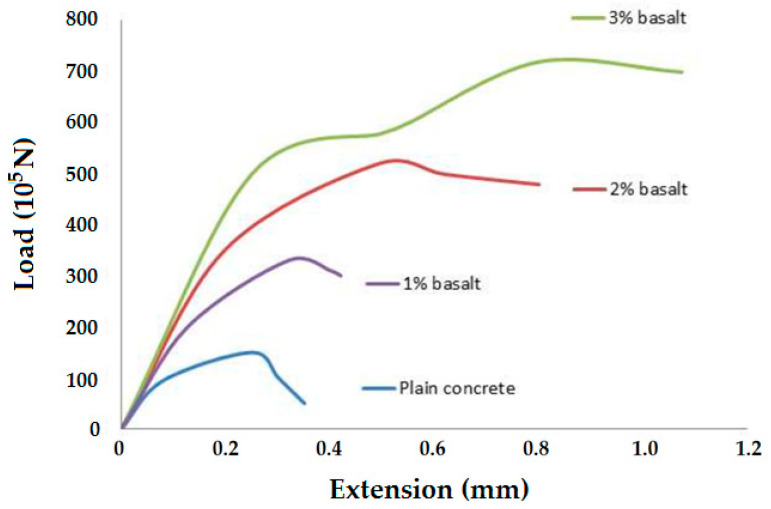
Tensile behavior of concrete reinforced with basalt fibers. Adapted from [20].

Basalt fiber, which is made from molten rock, exhibits excellent mechanical properties and exceptionally high heat resistance. It is relatively cheaper than carbon fiber and can be applied in thermal insulation, beams, reinforced concrete, and different types of automotive components. Concrete material in which basalt fibers are used shows very high heat resistance, lighter weight, and superior mechanical properties. Researchers have used basalt fiber in concrete and concluded that 2% of basalt fibers used in the overall composition of concrete could improve the flexural strength by 40 to 50% after 14 days of curing [17]. Compressive strength increases up to 92% after 28 days of curing concrete. They also suggested that the optimum time for mixing basalt fibers in the concrete mix should not be more than 1.5 minutes, otherwise the basalt fibers could segregate from the concrete mix. Another study found that the use of basalt fiber in concrete can increase the tensile strength and modulus of rupture [18]. Maximum strength was achieved when the volume percentage of basalt fiber was 1.5%. The modulus of rupture increases by 13 to 57%, and tensile strength increases by 9.6 to 22.58% when the volume fraction of basalt fibers increases to 1.5% [17,18,19]. The heat-conducting capacity of concrete decreases as the percentage of basalt fibers increases. Several researchers concluded that the bending and tensile strength of concrete increases by reinforcing basalt fibers in the concrete mix. The toughness and ductility of concrete increase with the addition of an optimum percentage of basalt fiber [18,19]. It has also been experimentally established that up to 3% basalt fiber reinforcement in concrete gradually improves the tensile performance [20]. The effect is shown in Figure 2.

The reinforcement of basalt fibers in the concrete mix can reduce the consumption of cement and affect the environment in a positive way. Basalt fiber is slightly more expensive than glass fiber. The potential application of basalt fiber is mostly in construction for anti-seismic buildings and structures that are exposed to extremely high pressure as well as temperature.

#### 3.1.3. Carbon Fiber

The fibers, which are composed solely of carbon atoms and have diameters of about 5 to 10 micrometers, are popularly known as carbon fibers. These fibers exhibit an extremely high ratio between their strengths and weight, very high tensile modulus, relatively lower thermal expansion, and high resistance to thermal degradation. The application of carbon fibers is mainly in aerospace, civil engineering, military products, and the sports sector. The cost of carbon fiber is much higher than that of other fibers like basalt, glass, Kevlar, and other polymeric fibers. The mechanical properties of the concrete material can be improved by using carbon fiber as reinforcement. Researchers have studied the change in mechanical performance of concrete when carbon fiber reinforcement was used [21]. They reported that an increase in the uniaxial tensile strength of 32, 48, and 56% can be achieved by adding 1, 2, and 3% of carbon fibers to a concrete mix. The flexural strength of concrete was also significantly improved by 72, 95, and 138% by adding 1, 2, and 3% of carbon fibers, respectively. The reliability of concrete under flexural loading increases with its reinforcement with carbon fiber [22]. Another study found that the use of 0.5% carbon fiber reinforcement in concrete resulted in an increase in the flexural strength by 85% and an increase in flexural toughness by 205% after curing for 28 days [23]. The flexural strength of the concrete increases by a minimum of 0.1% with the addition of carbon fiber. The optimum fiber length was 12 mm before mixing and 7 mm after mixing. Researchers used different percentages of carbon fibers in concrete and concluded that reinforcement of carbon fiber in concrete increased the compressive strength from 21 MPa to 47 MPa after 28 days of sample curing. The split tensile strength also increased significantly by the reinforcement of carbon fibers [24]. The addition of 1.5% carbon fiber in concrete showed superior properties. Carbon fibers are tightly bonded with concrete, and the failure of the concrete was divided into two steps: firstly, the fiber pullout, and secondly, the fiber breakage. Carbon fiber shows a higher tensile strength than steel fiber, and it is noncorrosive in nature. Therefore, it is more durable compared to steel fiber and proves to be a better replacement for steel fibers. Carbon fiber is slightly more expensive than steel fiber, but concrete reinforced with carbon fiber is lightweight and exhibits higher strength, flexibility, and durability compared with steel-reinforced concrete. The potential application of carbon fiber can be in complex building structures, repairing bridges, and high-strength building structures.

The concrete structures containing carbon fibers are durable, exhibit higher tensile strength, and therefore, can be successfully used for anti-seismic constructions. Concrete reinforced with carbon fibers can reduce the consumption of cement in the concrete mix and can be beneficial for the environment.

**Figure 3:**
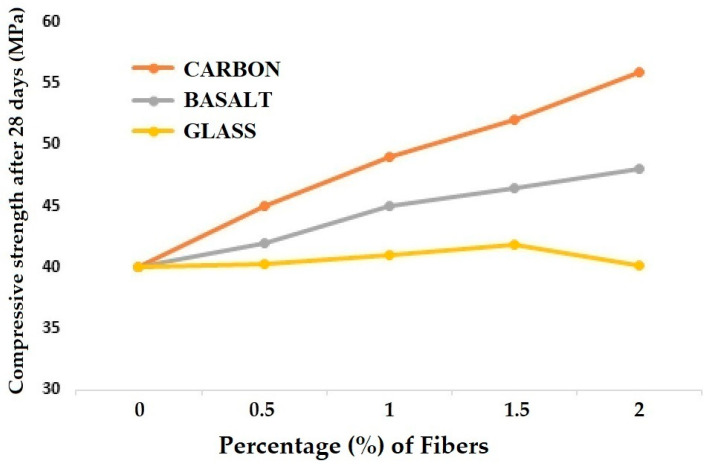
Compressive strength of concrete reinforced with high elastic modulus (HEM) fibers.

**Figure 4:**
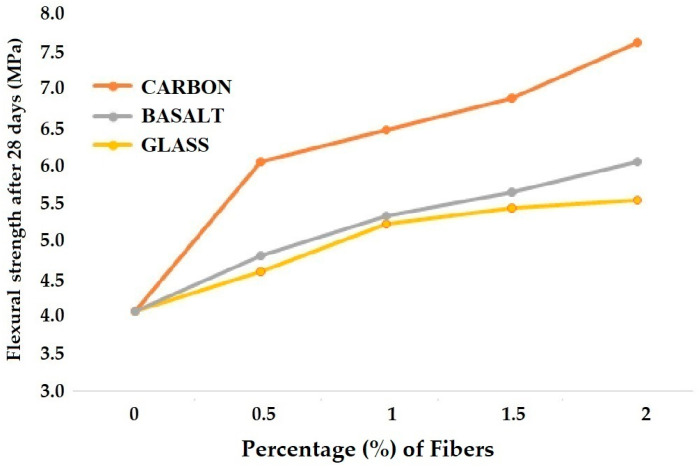
Flexural strength of concrete reinforced with high elastic modulus (HEM) fibers.

The comparison of compressive and flexural strengths for different varieties of concrete reinforced with high elastic modulus fibers is given in Figure 3 and Figure 4, respectively [15,18,24].

From Figure 3, it can be observed that the compressive strength of concrete gradually increases when the fiber loading increases from 0.5% to 1.5%. This holds good for carbon, basalt, as well as glass fiber-reinforced concrete samples. Further increase in carbon and basalt fiber loading to 2% seems to improve the compressive strength. However, increasing the glass fiber % to 2% decreases the compressive strength. It can be due to a lower strength and modulus of glass, which might lead to the brittle nature of concrete at higher fiber loading. The fiber mechanical properties seem to affect the compressive strength in a proportional manner. Therefore, the carbon fiber-reinforced concrete is better than the basalt-based concrete samples. The glass fiber-reinforced concrete samples show the minimum compressive strength among the three.

Figure 4 demonstrates the trends in flexural strength of HEM fiber-reinforced concrete when the fiber percentage varies from 0 to 2%. For all three types of HEM fiber-reinforced concrete, the flexural strength value increases when the fiber loading increases from 0.5% to 2%. The carbon fiber-reinforced concrete exhibited higher flexural strength than basalt fiber-reinforced concrete. Glass fiber-reinforced concrete showed the minimum flexural strength. Due to the lower mechanical performance of the glass fibers, the change from 1.5% to 2% of fiber loading is not as significant as in the case of carbon and basalt fibers.

### 3.2. Low Elastic Modulus (LEM) Fibers

Selected mechanical properties of LEM fibers used for reinforcement in the concrete mix are given in Table 2. The fibers included in LEM are both natural and manmade.

#### 3.2.1. Natural Fibers

**Figure 5:**
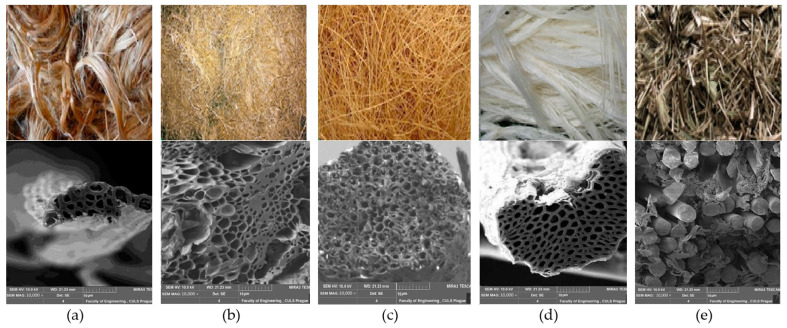
Natural origin fibers used for reinforcement in concrete: (**a**) Jute fiber, (**b**) Sugarcane/bagasse fiber, (**c**) Coconut/coir fiber, (**d**) Sisal fiber, (**e**) Basalt fiber. Adapted from [36].

The hair-like material, which can be derived/extracted from renewable natural sources like plants, animals, or minerals, is called a natural fiber. Natural plant-origin fibers include cotton, sisal, jute, hemp, banana, and coconut fibers. These fibers are used in building materials, insulation boards, clothing, cosmetics, and medicine. Due to their biodegradability and ease of availability, they contribute to a healthy ecosystem and a low-cost alternative that can fulfil the needs of the construction and building industries. Selected natural fibers, which are used as reinforcement in concrete, are shown in Figure 5 and discussed in the subsequent sections [33,34,35,36].

**Figure 6:**
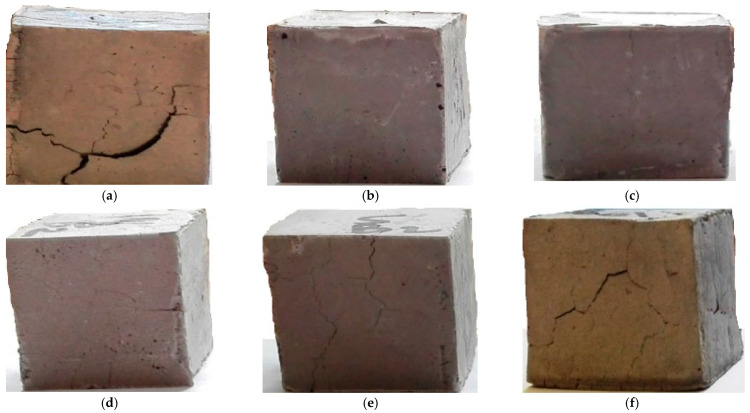
Concrete sample blocks after compression test and cracking, (**a**) Control sample, (**b**) Basalt fiber-reinforced, (**c**) Jute fiber-reinforced, (**d**) Sisal fiber-reinforced, (**e**) Coconut/coir fiber-reinforced, (**f**) Sugarcane/bagasse fiber-reinforced. Adapted from [32].

The sample concrete blocks created with reinforcement of selected natural-origin fibers can be seen in Figure 6.

##### Jute Fiber

**Figure 7:**
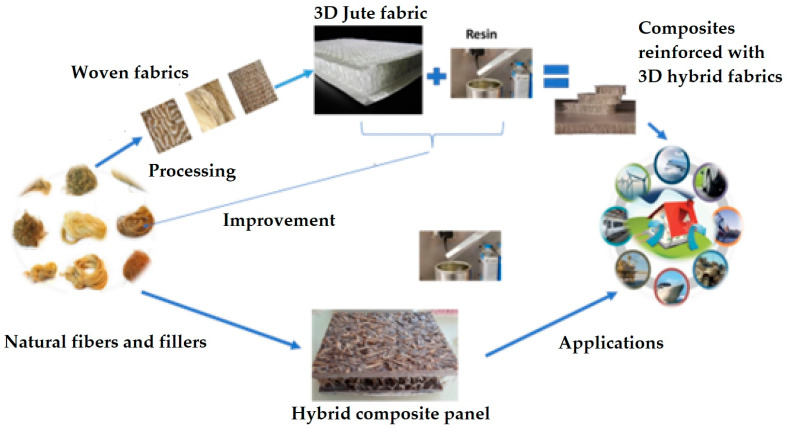
Examples of 3D structures from jute fibers as an alternative sustainable reinforcement material in lightweight constructions. Adapted from [41].

Jute is classified as a bast fiber, which is obtained from a flowering plant of the genus *Corchorus* and the family *Malvaceae*. It is the second most affordable and low-cost natural fiber. It shows relatively high tenacity, heat insulation, low thermal conductivity, and sound insulation properties. Jute fiber is used in ropes, curtains, carpets, clothing, and the construction industry. Researchers have investigated the durability and mechanical properties of different grades of concrete reinforced with jute fibers [34]. It has been reported in the literature that the workability of the concrete was reduced by the addition of jute fibers. When 1% jute fibers were added to the concrete mix, the maximum tensile strength and compressive strength were achieved. The fiber and matrix bond between jute and cement is very strong, which improves the mechanical performance of the concrete. However, in an acidic environment, there is no significant effect of jute fiber on the concrete. Several researchers have also reported using varying percentages and cut lengths of jute fibers in concrete and concluded that tensile, flexural, and compressive strengths of concrete increase significantly with the addition of 0.5 to 1.25% of jute fiber into the mix [35]. The tensile strength increases by 35% when compared with normal concrete. Hence, the reinforcement of the jute fiber in concrete significantly improves the mechanical performance of concrete, along with a reduction in the overall cost of the construction material [36,37]. It has been concluded that the chemically treated jute fiber shows 60 to 66% improvement in mechanical properties compared to untreated jute fibers [38,39,40]. The chemically treated jute fiber improves compressive strength by 11% and flexural strength by 86% compared with concrete without jute fiber [40]. Researchers have developed a technique to use hybrid 3D structures from jute fibers as an alternative sustainable reinforcement material in lightweight building constructions, as depicted in Figure 7 [41].

**Figure 8:**
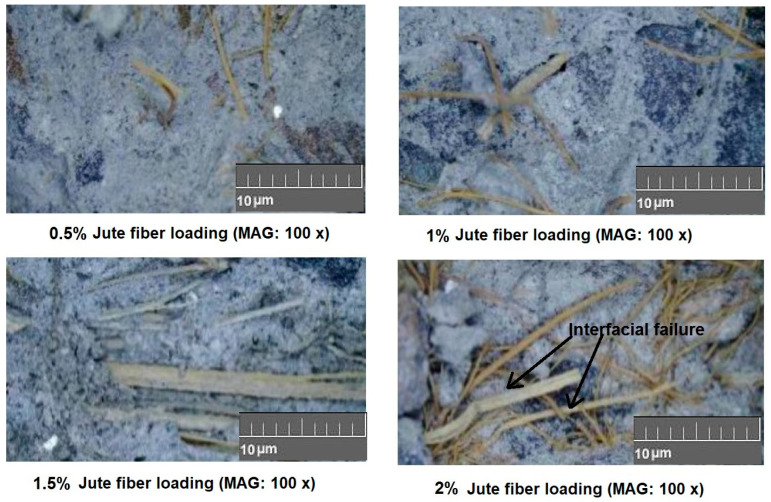
Fiber–matrix(cement) bonding while using various % of jute fibers in concrete. Adapted from [36].

Figure 8 shows images of fiber–matrix(cement) bonding while using various % of jute fibers in concrete [36].

The cracked concrete samples after the bending test are shown in Figure 8 and were analyzed optically from photographic images. It can be observed that an increase in fiber percentage beyond 1.5% leads to crack initiation and detachment of fibers from the cement matrix [36,41].

##### Sisal Fiber

Sisal is a type of natural fiber obtained from the plant Agave sisalana. They are flexible, durable, and have the potential to stretch and exhibit significant resistance against degradation in saline water. Sisal fiber is used in making clothes, ropes, footwear, bags, and geotextiles. These can also be used as reinforcement for composite and concrete. Researchers used different percentages of sisal fibers in concrete and reported that flexural, compressive, and split tensile strength of the concrete increased when sisal fiber reinforcement was used in the concrete mix [42]. The increase in compression strength was found to be maximum at 1% fiber content, and for split tensile strength, it was possible with the addition of 1.5% fiber. There was a decrease in workability of the concrete by the subsequent increase in the percentage of sisal fibers. The crack resistance and flexural strength of the concrete samples reinforced with 1% sisal fibers also increased significantly. It was found that the increase in compression strength is about 28% when the percentage of sisal fiber in construction materials is 1% [43]. The flexural strength and split tensile strength of concrete also increased by 31.13% and 20.1%, respectively, when the volume fraction of sisal fiber in concrete was 2%. The effective percentage of sisal fiber in concrete is, therefore, optimized between 1 and 2% [44]. It was found that when different fractions of sisal fibers are used in M20 grade concrete, the compressive strength increases by 18.17% at 1 wt% of the sisal fiber compared with normal concrete [45].

##### Banana Fiber

Banana fiber is a type of natural fiber that can be extracted from the pseudo-stem or layers of bark of the banana plants. These are lightweight, biodegradable, fire-resistant fibers with relatively lower elongation at break and higher specific strength. Applications of banana fibers include making ropes, high-quality paper cards, paper for currency notes, high-quality fabrics, textiles, and construction materials. Banana fibers can also be considered to partially replace some components in the concrete mix because of the promising mechanical properties. Researchers have used banana fibers in concrete and concluded that the addition of banana fiber increases the tensile and compressive strengths of the resultant concrete [46,47]. There was a 45.04% increase in compressive strength compared to normal concrete with the addition of 2% banana fiber to the mix. The tensile strength increased by 47.39% with the addition of 2% banana fiber compared with pure concrete. However, the tensile strength further decreases when the percentage of fiber is increased above 2%. Fibers are added to concrete to improve resistance to cracking or, sometimes, to reduce its cost. Researchers have used banana fibers in concrete and concluded that a small percentage of banana fiber, up to 2%, actually improves strength and the overall performance of concrete [48]. But the compression strength decreases when the fiber percentage increases above 2%. Accumulation of the fiber or formation of the fiber ball in concrete occurs when the fiber percentage increases above the optimum level, which might lead to deterioration in some properties of the concrete. For a sustainable and eco-friendly green construction material, researchers have even used banana fiber bars in concrete [49]. It was concluded that by adding 2% banana fiber to concrete, the flexural strength increases by 45%. It has also been reported that the maximum capacity for load bearing at failure can be significantly improved by adding banana fibers up to 2% [50].

#### 3.2.2. Synthetic Fibers

The man-made fibers, which are produced by chemical reactions, are called synthetic fibers. Examples are polyester, polypropylene, polyamide (nylon), polyethylene, etc.

##### Polyester Fiber

Several researchers have used polyester fibers and recycled particles/chips from polyethylene terephthalate (PET) in concrete [26,51,52]. It was concluded that the workability of the concrete mixture decreased with the reinforcement of the polyester fibers. However, improvements in the tensile, flexural, and compressive strength were observed in concrete reinforced with 1% polyester fibers. There was a 21% increase in compressive strength, 15% increase in tensile strength, and 16% increase in flexural strength when 1% PET particles were added. Different percentages of polyester fibers were used in concrete to optimize flexural, compression, and tensile strengths. All such mechanical properties improved by using 1% polyester fiber or PET particles in concrete. Further, the ductility increased, and excellent crack resistance was obtained at 1% fiber content [52,53].

##### Polyamide (Nylon) Fiber

The durability of nylon fiber-reinforced concrete has been extensively studied. It has been reported that the durability of concrete increases at an optimum limit of nylon fiber, i.e., 0.5% [54]. The addition of 0.5% nylon fiber to concrete protects the concrete structure from any hazardous situation and improves the overall service life and durability of the concrete [55]. It was also concluded that the addition of 0.5% nylon fiber to concrete shows a positive effect on compressive and tensile strengths [56]. When the percentage of nylon fiber is increased above 0.5%, the compressive strength starts decreasing as the porosity of the concrete increases. The reinforcement of concrete with nylon fibers increases the plasticity and delays the crack propagation in the structure [57,58].

##### Polypropylene (PP) Fiber

Polypropylene (PP) fibers were extensively used as reinforcement in concrete to improve mechanical strength and performance [28,29]. It has been reported that the PP fiber in concrete increases the compressive strength and tensile performance [59]. After 7 days and 28 days of curing, the compressive strength of the concrete can be increased by 20% and 16%, respectively. Similarly, the split tensile strength of the concrete increased by 11% and 17% after 7 days and 28 days of curing. The optimum percentage of PP fibers as partial replacement of cement in concrete was found to be 1.5% [59,60]. Beyond this amount, the strength of concrete starts decreasing [60,61]. Different percentages of PP fibers were used in concrete, and it was found that as the fiber % in concrete increases, the flexural and compressive strengths of concrete start to decrease. This is because of the comparatively inferior mechanical performance of polypropylene fibers and a relatively weaker bond strength with cement. There was an increase in the water absorption capacity of concrete by increasing the dosage of polypropylene fiber above 1.5%, indicating higher porosity [62]. That may be another reason for decreasing mechanical performance.

##### High-Density Polyethylene (HDPE)

High-density polyethylene (HDPE) is a thermoplastic polymeric fiber produced from the polymerization of ethylene monomers. HDPE is durable, lightweight, chemical-resistant, and shows high tensile strength. Applications of high-density polyethylene are in the chemical industry, plastic industry, construction industry, outdoor and indoor sports grounds, wire and cable insulation, and homeware. HDPE is used in concrete because of its good mechanical and chemical properties. Researchers used recycled high-density polyethylene in concrete and studied its mechanical performance [63]. There was a 3 to 14% increase in tensile strength, though the elastic modulus and compressive strength were not affected significantly by the addition of high-density polyethylene fibers in concrete. This can be attributed to the very high extensibility of HDPE fibers. When 0.5 to 2% of HDPE fibers were added to concrete, it increased the flexural toughness, reduced the plastic shrinkage, and increased the permeability for water within concrete. The concrete reinforced with HDPE proved to be more durable than conventional concrete [64]. It was concluded that there is a marginal increase in the compressive strength of concrete reinforced with 0.5–1.5% fibers with respect to the mass of concrete [64]. When the fiber loading was further increased beyond 1.5%, there was a subsequent decrease in the compressive strength. The tensile and flexural strengths were increased by the addition of HDPE fibers in concrete. The tensile strength increased from 2.9 to 5.2 MPa, while the flexural strength increased from 1.8 to 2.1 MPa by increasing fiber dosage to 2% [65]. It was concluded that different percentages of recycled HDPE fibers used as reinforcement in concrete can result in about a 39.14% increase in the splitting tensile strength and 36.22% increase in flexural strength after 28 days of curing of the sample. The fibers form strong bonding with the surrounding matrix (cement), which increases the resistance to crack propagation [66]. HDPE significantly reduces the crack formation and plastic shrinkage in concrete.

**Figure 9:**
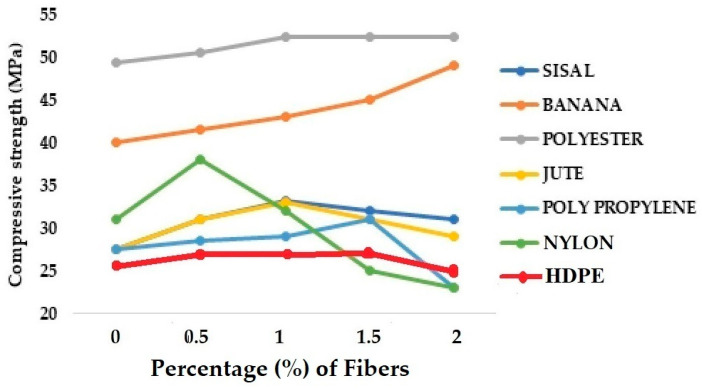
Compressive strength for different grades of concrete samples reinforced with 0 to 2% of natural and synthetic (LEM) fibers.

The comparison of compressive strength for different grades of concrete samples reinforced by 0 to 2% of natural and synthetic fibers is shown in Figure 9 [34,36,41,43,47,51,54,62,65].

From Figure 9, it can be observed that the different natural and synthetic fibers show different behavior when used as reinforcement in concrete. Jute and sisal are relatively denser cellulosic fibers compared to banana. The concrete reinforced with jute or sisal fibers has almost the same compression strength. The maximum strength was observed at 1% fiber content. As the fiber content increases from 1 to 1.5 and then to 2%, the compressive strength further decreases owing to higher elongation of these fibers compared to banana fibers. The compressive strength of banana fiber-reinforced concrete was higher than that of jute and sisal fiber-reinforced concrete. Also, banana fiber content up to 2% resulted in a gradual increase in compressive strength. It is understood from the data in Table 2 that banana fiber has a higher elastic modulus and lower extension than jute and sisal fibers. That may be responsible for improved compressive strength.

Among the synthetic fibers used, polyester fibers are the strongest. Therefore, the influence of using polyester fiber as reinforcement in the concrete on the compressive strength is clearly visible. The optimum amount of synthetic fiber ranges between 0.5 and 1%. Nylon fiber-reinforced concrete shows a decline in compressive strength when the fiber loading increases beyond 0.5%. There seems to be poor adhesion between nylon fibers and concrete. Both Polypropylene and HDPE fiber-reinforced concrete result in the maximum possible compressive strength at about 1.5% dosage.

**Figure 10:**
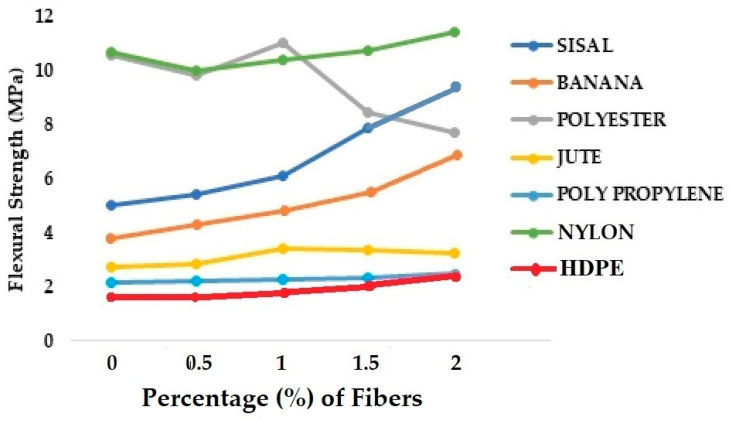
Flexural strength for different grades of concrete samples reinforced with 0 to 2% of natural and synthetic (LEM) fibers.

Figure 10 shows the trends in flexural performance of concrete samples reinforced with 0 to 2% of LEM fibers [34,36,41,43,47,51,54,62,65].

A slightly different trend can be observed in Figure 10. An increase in the percentage of fiber content seems to improve the flexural performance of concrete in most cases, except for polyester fibers. There is an increase in the flexural strength of polyester fiber-reinforced concrete at 1% fiber content. Beyond this, the fibers do not bond well with cement, and there can be occasional fiber slippage. These may not affect the compression behavior, but may significantly affect the flexural strength.

## 4. Various Types of Waste Fibers Used as Reinforcement in Concrete

Large amounts of waste are produced by industrial, agricultural, and domestic activities. The production and accumulation of waste materials cause significant environmental pollution. The waste materials can be either biodegradable or non-biodegradable. Biodegradable waste is the type that can be broken down by microorganisms into CO_2_, water, methane, and organic compounds by natural processes, while non-biodegradable waste is the type that cannot be decomposed by natural processes.

### 4.1. Non-Biodegradable Waste

These are the waste materials that cannot be decomposed by bacterial, fungal, or viral activities in nature and remain undecayed on Earth for thousands of years. These types of waste materials typically cause major environmental issues. Plastic waste and metallic waste are types of non-biodegradable waste. The application of such non-biodegradable waste materials in concrete can improve the ductility and reduce the disposal of waste as landfill in the environment.

#### 4.1.1. Different Types of Polymeric/Textile Wastes

**Figure 11:**
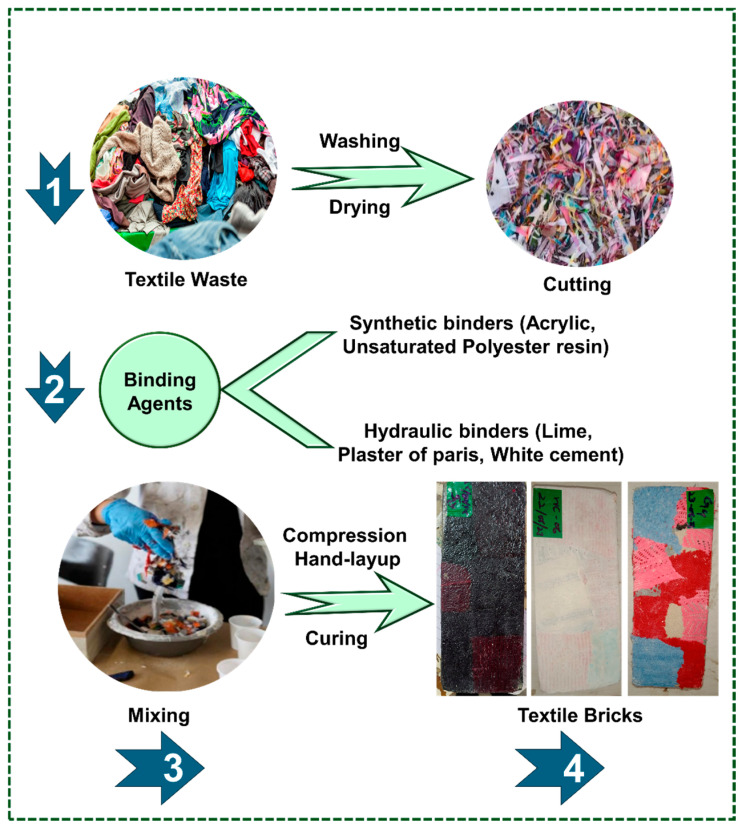
Construction materials developed from textile waste as reinforcement in concrete. Adapted from [7].

The possibility of using various types of textile waste as reinforcement in concrete has been studied by different researchers [4,6,7,67,68,69,70,71]. It has been reported that the use of polymeric/textile waste in concrete can reduce the solid waste in the environment while improving the overall mechanical performance of concrete. Simultaneously, it can help reduce the overall cost of construction material [69,70,71]. The inclusion of 0.5% waste of textile/polymeric materials in concrete shows improved results, while larger dosage increases voids, which result in reduced mechanical performance. The addition of polypropylene waste in concrete subdivided the larger pores into smaller pores, which increases the compressive strength and permeability for water inside concrete [13,20]. Waste of nylon fibers can enable a strong fiber and matrix (cement) bond, which results in enhanced flexural strength or bending stiffness of concrete. Thus, textile waste materials can be used as an alternative for aggregates for reinforcement in concrete in order to reduce the environmental pollution and cost of concrete [67,68,69,70,71]. A schematic for recycling textile waste for construction materials is shown in Figure 11 [7].

Different types of metallic fiber waste were used together with polymeric fibers, and several mechanical performances of the concrete, e.g., compressive strength, ductility, flexural strength, and toughness, were investigated [72,73,74,75,76]. It was found that by adding up to 1.5% metallic waste, there was no significant effect on compressive strength, but when the amount of metallic waste fibers in concrete increased to 2%, there was a decrease in the compressive strength. Interestingly, the combination of metallic waste fiber and polypropylene waste fiber in concrete increases the crack resistance and ductility of concrete [73]. The performance of PET waste as a replacement for fine aggregates in concrete was investigated, and it was reported that the addition of PET waste as fine aggregates increases the compressive strength of concrete [12]. By using 10% PET waste as reinforcement in concrete, there was an 11% increase in compressive strength. The addition of PET to concrete also increased the split tensile strength. There was a 15% increase in flexural strength when fine aggregate was replaced by 10% PET waste/powder. It was concluded that concrete with PET waste can be used in low-strength applications [51,53]. In the construction industry, researchers are focusing on providing cost-effective alternative materials with increasing strength. Many attempts have been made to use different types of waste materials in concrete to reduce cost and improve strength. Different types of waste, e.g., lathe waste, soft drinks bottle caps, empty tin waste in the form of strips, and workshop steel waste in the form of powder, were used in concrete at a dosage of 1% [53]. The compressive strength of concrete made with steel powder showed an increase of 41% and the tensile strength increased by 40%. The strips of soft drink bottle caps, when used in concrete, increased the flexural strength by 25.88% [12,51].

Several researchers used recycled polypropylene fiber from the carpet industry in concrete and investigated its influence on split tensile strength, flexural strength, compressive strength, and shrinkage [12,29,59]. There was an increase in compressive strength of concrete by the addition of 0.5% polypropylene waste fibers, but when the fiber percentage was increased to 1%, the compressive strength decreased. Optimum split tensile strength was achieved when the fiber percentage in concrete was limited to 1%.

**Figure 12:**
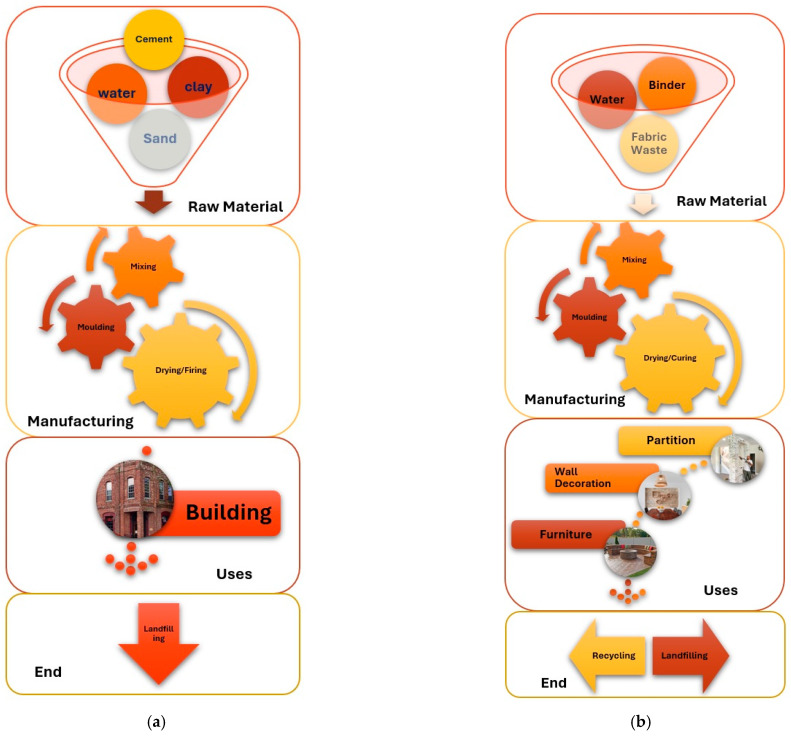
Life cycle analysis of (**a**) standard concrete and (**b**) concrete reinforced with recycled textile waste. Adapted from [7].

A life cycle analysis of textile waste-based construction and standard construction is shown in Figure 12 [7].

Researchers have tried to use waste masks in concrete to produce lightweight, environmentally friendly green concrete. In addition, the use of facial masks in concrete solves the waste management problem. By including a 0.5% facial mask fiber in concrete, the compressive strength increased by 8.3% [77]. When the amount of face mask waste in the concrete increased beyond 1%, both tensile and compressive strength decreased. The addition of 1% mask waste to concrete reduces its permeability compared to normal concrete and, hence, improves the water resistance of concrete. The ideal percentage of waste masks in concrete, which greatly influences the overall performance, was found to be 0.5% [77,78,79]. The water-absorbing capacity of concrete increases by using recycled mask as reinforcement. The addition of waste masks significantly improved the overall quality of concrete compared with conventional concrete. The concrete containing waste masks exhibits improved crack resistance and modulus of elasticity [79].

**Figure 13:**
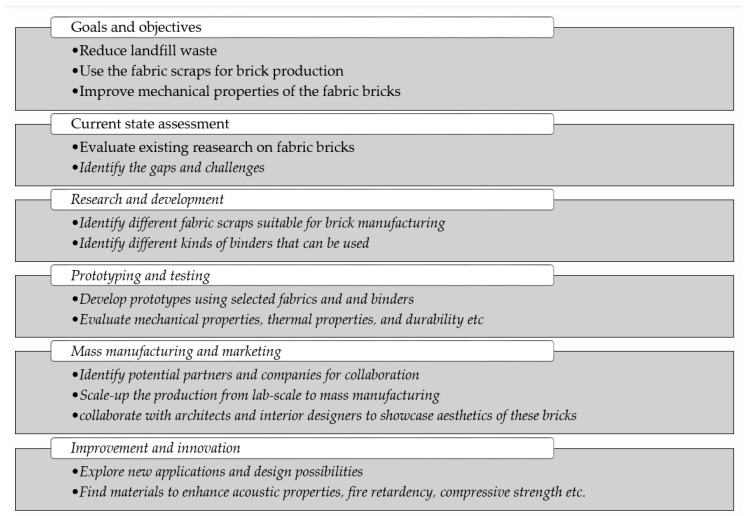
A roadmap for sustainable construction materials by using recycled textile waste. Adapted from [7].

A road map for the utilization of polymeric/textile waste in construction materials has been proposed and shown in Figure 13 [7].

#### 4.1.2. Steel Waste Fibers

In recent years, the possibility of applying steel fibers in concrete to improve the mechanical properties has been thoroughly investigated. Researchers have investigated how using clean recycled steel fibers (CRSF) and recycled steel fibers with impurities (RISF) in concrete can affect its mechanical and economic performance at room and higher temperatures [80,81,82,83]. Standard concrete was also compared with industrial steel fiber-reinforced concrete. Results indicate that CRSF-reinforced concrete performed similarly to concrete reinforced by industrial steel fiber and better than standard concrete at room temperature and 200 °C. However, RISF-reinforced concrete exhibited lower mechanical performance due to impurities reducing the bond strength. Interestingly, at 600 °C, RISF showed improved results compared to 200 °C. Based on the mechanical and cost analysis, CRSF could be a great alternative for reinforcing concrete structures [83,84,85].

This study indicates that the slump of concrete is reduced by the inclusion of recycled steel fibers and shows a decreasing trend with an increase in the fiber content. The compressive strength improves significantly by adding 1% primary steel fibers to concrete compared with concrete reinforced with recycled steel fibers or normal concrete [84,85]. The split tensile strength and flexural strength of recycled steel fiber-reinforced concrete and primary steel fiber-reinforced concrete are greater than those of plain concrete. The toughness of the concrete sample was significantly increased by adding recycled steel fibers. Recycled steel fibers can be used as an alternative to primary steel fibers in concrete for building constructions. The maximum percentage of recycled steel fibers should not be more than 1% in the concrete mix [85].

### 4.2. Biodegradable Waste Materials

The waste produced from plants and animals, and the waste that can be degraded by natural processes, is called biodegradable waste. Mostly, incinerated biomass waste is disposed of in landfills, without any control, which is causing pollution in the environment and is harmful to human health. The agricultural biomass can be mixed with construction material to address this issue. Both coarse and fine aggregates show very good affinity toward agricultural biomass and, thus, improve their mechanical characteristics.

#### 4.2.1. Coconut Waste Fibers

Coconut fiber or coir fiber, obtained from the outer cover of the fruit, is a very strong and durable reinforcement material. Coconut fibers are renewable, biodegradable, and possess excellent strength, extensibility, and high resistance to sunlight and saline water. The split tensile strength, flexural strength, and compressive strength of concrete reinforced by different ratios of coir fiber have been evaluated [69,70,71]. It has been concluded that when the percentage of coconut fiber increases from 1 to 3%, the tensile, flexural, and compressive strengths of concrete increase. From the reported literature, it is evident that concrete reinforced with coconut fiber is more effective than standard concrete [86,87]. Different percentages of coconut fiber were used in concrete, and the effect of high temperatures on mechanical performance was studied. At very high temperatures, the compressive strength of concrete increases with reinforcement with coconut fibers [88]. Coconut fiber also increases the ductility of concrete and reduces the overall cost of concrete structures [89]. Steel is normally used in concrete for reinforcement, but due to its higher cost, higher weight-to-strength ratio, and corrosive nature, the research is more focused on low-cost and non-corrosive reinforcements, e.g., coir fibers. Researchers have used different percentages of coir fibers to reinforce concrete and compared the cost, thermal conductivity, and strength with ordinary concrete [87,88,89]. It was concluded that the inclusion of coir fiber as reinforcement in concrete increases the thermal insulation of the buildings, and the concrete buildings reinforced with coconut fibers can lower the cost involved [30]. This study found that the flexural and compressive strengths of concrete can be increased when the percentage of coconut fiber increases to 3%. The split tensile strength of concrete reaches a maximum at 3% fiber content and gradually decreases above 3% [87,88]. When the fiber percentage is lower than the fine-sized aggregates, they enter into the surface pores and form a perfect bonding, which increases the compressive strength of concrete, but when the fiber percentage increases, the bonding between the fibers and cement does not occur efficiently and may decrease the compressive strength. The optimum percentage of coconut fiber in concrete for overall effectiveness is 2–3% [89,90,91]. It was concluded that the toughness and tensile strength of concrete increase with the addition of coconut fiber if the percentage of reinforcement is less than 3%. The inclusion of coconut fibers in concrete increases the resistance to cracking and the ability of the structure to efficiently absorb more energy [90]. It was observed that plain concrete fully crashed when its failure load was reached, but concrete with 1 to 3% coconut fiber as reinforcement resisted its crash even after applying the ultimate failure load [91].

#### 4.2.2. Sugarcane Waste Fibers

Researchers have used different percentages of sugarcane waste fibers, otherwise known as bagasse, in concrete for sustainability and to improve the mechanical properties [92]. They concluded that by using 0.5–1.5% sugarcane fiber, the compressive strength can be increased. Above this percentage, there is a decrease in compressive strength of concrete. The optimum tensile strength was obtained at 1.0% of sugarcane fiber in lightweight concretes [93,94,95]. Different ratios of sugarcane waste fibers were used in concrete as a replacement for cement, and it was found to reduce the width of the cracks [96,97,98]. By using 1% sugarcane waste fibers as reinforcement in concrete, the compressive strength can be increased by 20%, and the split tensile strength can be increased by 33%. When the percentage of sugarcane waste fiber increases above 2%, the mechanical performance of the concrete starts deteriorating [98,99,100,101,102].

#### 4.2.3. Rice Husk

Several researchers partially replaced the cement with different amounts of ash produced from rice husk [103,104,105,106]. They concluded that by adding 1.5% rice husk as a replacement for cement, the compressive strength can be increased by 6.8%, and tensile strength can be increased by 7.2% after 28 days of curing [103,104]. The inclusion of rice husks as fillers in concrete can reduce the water permeation by 26% and chlorine permeation by 76% [107,108,109,110]. The reinforcement of concrete by rice husk ash can significantly reduce the overall density of concrete [111,112,113]. It was found that the addition of ash from rice husk as reinforcement/filler in concrete reduces the consumption of cement and reduces the weight of construction material without compromising the overall mechanical performance [110,113].

#### 4.2.4. Wheat Straw

Researchers used different ratios of wheat straw in concrete as reinforcement and compared its properties with normal concrete [114,115,116,117,118]. The addition of 1%, 2%, and 3% wheat straw to concrete reduced the compressive strength by14%, 27%, and 37%, respectively [119,120]. When wheat straw percentage increased, the energy-absorbing capacity of concrete decreased [36,121].

#### 4.2.5. Bamboo Waste Fiber

**Figure 14:**
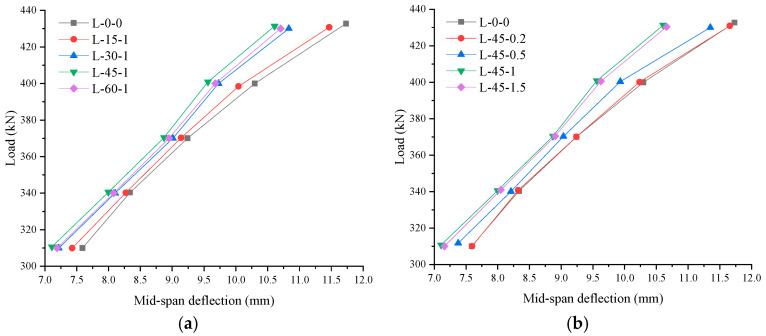
Load–deflection relationship of concrete beams reinforced with bamboo fibers: (**a**) effect of fiber length; (**b**) effect of fiber percentage. Adapted from [126].

Bamboo waste fiber (BWF) offers several advantages, like eco-friendliness, renewability, cost benefits, and advanced mechanical performance, making it a promising reinforcement material in order to improve the brittleness and durability of concrete. Several investigations have reported a lot of information on BWF-reinforced concrete for lightweight constructions [122,123,124,125]. The effects of bamboo fiber content and its length on the compressive performance, modes of failure, and load-elongation curves were studied. BWFs efficiently suppress the initiation and propagation of internal cracks in concrete, thus playing a crucial role in resistance to rupture and enhancement of fracture toughness. In addition, the splitting tensile strength of concrete can be improved significantly. Though a slight decrease in compressive strength was observed by increasing the bamboo fiber content beyond 1.5% [126,127]. Figure 14a,b shows load–deflection relationship diagrams for concrete beams reinforced with bamboo fibers of different lengths and fiber volume percentage [126].

By maintaining the same content of bamboo fibers, it was found that the fiber length did not affect the load elongation behavior significantly. The fiber content affects the uniaxial load-bearing capacity. There was an efficient transfer of force between the cement and fibers, which depends on the distribution density of fibers. The overall mechanical performance of bamboo fiber-reinforced concrete is affected by the fiber orientation and distribution [126].

**Figure 15:**
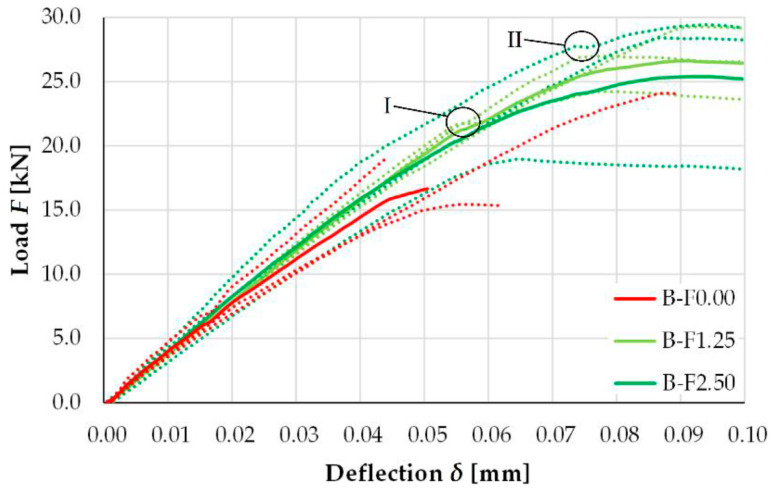
Load–deflection curves of concrete reinforced with bamboo fiber between 1.25% and 2.5% fiber loadings. Adapted from [127].

Further, Figure 15 shows load defection curves, depicting the effect of bamboo fiber percentages varying between 1.25% and 2.5% [127].

In Figure 15, the individual sample measurements are shown by the dotted lines, and the average result is represented by the solid lines of the test series at a given deflection. It can be seen that the load–deflection behavior of the concrete beams without any reinforcement of bamboo fibers (B-F0.00) shows a linear progression until the maximum force of 19.5 kN. The curves of the bamboo fiber-reinforced concrete beams show higher maximum force than the plain concrete beams. Thus, the breaking load of the sample B-F1.25 was 26.8 kN, which is 37.1% higher than the normal beam. The breaking load for sample B-F2.50 was 25.6 kN, and it was 31.3% higher than that of the normal unreinforced beam. For the unreinforced concrete beams, the load–deflection curves were always linear till break. But for the bamboo fiber-reinforced concrete beams, they were linear only up to a certain point before the actual failure occurred. In these samples, a drop in strength was observed, which can be seen as a kink in the latter part of the curves (depicted by I in Figure 15). In some other samples, there was a drop in strength right before the maximum force was reached, i.e., before the failure (depicted by II in Figure 15) [127].

#### 4.2.6. Flax and Hemp Waste Fibers

The possibility of using flax and hemp waste fibers as a sustainable reinforcement in concrete in order to replace synthetic filler materials/fibers is studied extensively by researchers [128,129,130]. The main problem with plant-origin fibers is the large variations in their properties and a relatively higher biodegradability under alkaline pH conditions in the concrete mixture [128,129,130].

**Figure 16:**
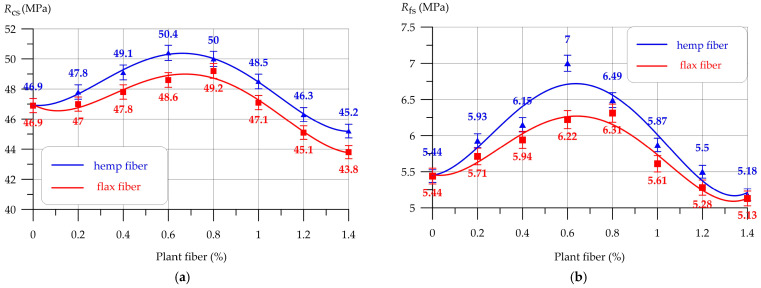
Comparative analysis of concrete beams reinforced with flax and hemp fibers: (**a**) compressive strength (*R_cs_*); (**b**) flexural strength (*R_fs_*). Adapted from [133].

Extensive research has been carried out to understand the application of bast fibers, e.g., hemp and flax, as reinforcement of concrete. Researchers have compared their techno-economic viability under practical conditions for construction purposes. The optimum percentage of hemp fiber as reinforcement in concrete was reported to be 0.6%. This resulted in a 7.46% increase in compressive strength and a 28.68% increase in flexural strength. On the other hand, the optimum percentage of flax fiber as reinforcement in concrete was found to be 0.8%. This showed a 4.90% increase in compressive strength and a 15.99% increase in flexural strength. The results proved that flax and hemp fibers can be used efficiently for reinforcement of concrete in practical building applications [130,131,132,133]. The comparative results of compressive strength and flexural strength in concrete beams reinforced with flax and hemp fibers of different percentages are shown in Figure 16a,b, respectively [133].

The limitations of including small dosages of flax and hemp fibers in concrete depend on the technological parameters needed for the preparation of such plant-origin fibers to be used as reinforcement in concrete. They need very strong alkalis for the removal of impurities and lignin. Further, plasticizing additives are needed to maintain the slump of the concrete mixture. The new concept of developing flax and/or hemp fiber-reinforced concrete is a positive step toward achieving the goals of sustainable constructions in the future [130,131,132,133]. These fibers have a relatively higher resistance to alkali and can survive under harsh environmental conditions. At the same time, they are biodegradable and eco-friendly. They can be promising alternatives to synthetic fibers in concrete, especially for construction works where resistance to aggressive chemicals is essential. They can also be used for repairing concrete structures, e.g., walls, columns, beams, bridges, etc. It is, however, necessary to optimize the durability of such concrete reinforced by flax and hemp fibers before selecting appropriate application areas. The cost-benefit analysis can provide further understanding of the suitability of such new material options in the construction sector [130,131,132,133].

#### 4.2.7. Kenaf Waste Fiber

The possible use of kenaf waste fibers to improve the mechanical properties of concrete has attracted several researchers. Adding kenaf fibers as reinforcement in concrete is an eco-friendly way to replace synthetic fibers [134]. The findings from experimental research showed that the mechanical performance of concrete reinforced with kenaf fiber shows several improvements. Adequate mixing proportions and procedures must be selected for producing kenaf fiber-reinforced concrete. Several investigators have compared the compressive strength, compressive modulus, modulus of rupture, and splitting tensile strength of concrete samples reinforced with kenaf fibers with those of normal concrete [135].

The findings show that the mechanical performance of kenaf fiber-reinforced concrete was slightly better than plain control concrete specimens, especially with 2% fiber content. Kenaf fibers help in the wider distribution of cracks in the concrete rather than bigger crack formation, resulting in a higher toughness in comparison with plain concrete [136]. The bond between the fibers and cement matrix was found to be strong enough to resist fiber slippage or matrix cracking [137]. Using such waste fibers from kenaf as reinforcement in concrete is extremely helpful in avoiding their disposal at the cost of environmental pollution.

A comparative summary of the properties of selected biodegradable fibrous waste used in concrete is given in Table 3.

**Figure 17:**
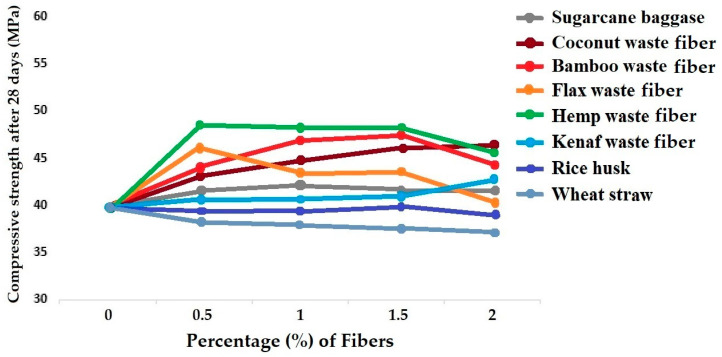
Comparative account of compressive strength for concrete reinforced with different types of biodegradable waste fibers and agrowastes.

Figure 17 shows a comparison of compressive strength for concrete samples reinforced with different types of biodegradable waste fibers and agrowastes.

It can be seen from Figure 17 that in most cases, the compressive strength of concrete improves with the inclusion of biodegradable waste fibers. Hemp and flax fibers provided the maximum improvement in compressive strength at 0.5% dosage. However, with a subsequent increase in fiber percentage, the compressive strengths decreased. This may be attributed to the overload of fibers and clustering, leading to some voids. Beyond 1.5% fiber content, there is a significant loss of compressive strength. Coconut fiber showed a steady increment of concrete compressive strength up to 2% loading. It is a result of an excellent fiber–cement interface and stronger bonding. Using 1.5% bamboo fiber waste as reinforcement in concrete also improves compressive strength by about 25%.

In the case of sugarcane bagasse, there was an increase in compressive strength by including 0.5–1% fiber in the concrete mix. However, a subsequent increase in fiber dosage can decrease the compressive strength due to poor fiber strength and bonding with cement. The inclusion of kenaf fiber waste also slightly increases the compressive performance up to 2% fiber content. Agrowaste (biomass) materials, e.g., rice husk and wheat straw, do not have a significant impact on the compressive strength. There is a small increase in compressive strength when 1.5% rice husk is used in concrete. On the other hand, the use of wheat straw actually reduces the overall compressive performance of concrete.

**Figure 18:**
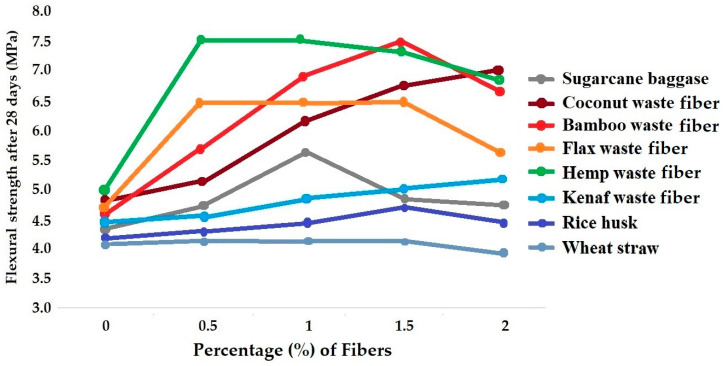
Comparative account of flexural strength for concrete reinforced with different types of biodegradable waste fibers and agrowastes.

Figure 18 shows the comparison of flexural strength for concrete samples reinforced with different types of biodegradable waste fibers and agrowastes.

Hemp and bamboo waste fibers provide the best flexural strength among all the different fiber types compared. However, the maximum flexural strength was achieved at different fiber contents. While 0.5% of hemp fiber is optimum for the best flexural performance, in the case of bamboo waste fibers, it was achieved with a 1.5% dosage. Flax fiber also gives the best results at 0.5% content. In the case of sugarcane baggasse, 1% fiber content resulted in the maximum flexural strength of concrete. As usual, coconut fiber/coir waste helps improve the flexural performance steadily up to 2% fiber content, indicating excellent fiber–cement interaction. Kenaf fiber also helps improve the flexural strength up to 2% content. Among the agrowaste (biomass) materials, rice husk slightly increased the flexural strength up to 1.5% but wheat straw failed to provide any improvement in the flexural performance. However, their contribution toward reducing the weight of the concrete cannot be ignored.

**Figure 19:**
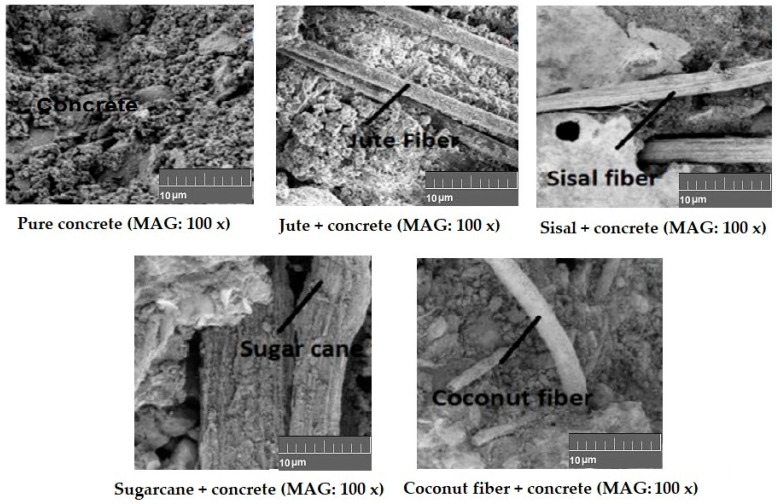
Interfacial bond between selected biodegradable waste fibrous materials and concrete. Adapted from [36].

Figure 19 shows the interfacial bond between selected biodegradable waste fibrous materials and concrete [36].

For all the concrete samples reinforced with natural-origin fibers, the ultimate tensile strength is higher than that of the control sample. With up to 2% fiber content, the tensile properties can be improved. Further increase in fiber content to 2.5% or 3% results in deterioration of tensile performance [36]. The behavior of different fibers and their waste materials in concrete is largely governed by their composition, the nature of the fiber surface, interfacial interaction with the concrete, and uniformity of dispersion in the concrete. The optimum fiber content is found in the range of 0.5–2%. This has to be considered on a case-by-case basis. Looking into these performance indicators, sometimes a combination of different fiber materials or their wastes may also be tried for reinforcement in concrete.

## 5. Conclusions

The following conclusions can be made based on the literature and analysis of the findings:

1. The concrete containing glass fiber is more durable, non-corrosive, lightweight, and cost-effective for high-performance applications. Glass fiber can be used as a partial replacement of cement with an optimum content of 1%. The potential application of glass fiber can be used in exterior building structures;
2. Basalt fiber increases the strength, ductility, and thermal resistance of concrete. The addition of basalt fiber to partially reduce cement consumption affects the environment in a positive way. The potential application of basalt fiber in construction is for anti-seismic buildings and buildings that are exposed to high pressure/temperatures;
3. Carbon fiber-reinforced concrete is durable, has high strength, and can be used for anti-seismic constructions. However, it is more expensive than conventional concrete;
4. Natural fibers are low-cost and easily available alternatives for replacing certain components in concrete. Concrete reinforced with natural-origin fibers, e.g., jute, sisal, and banana, shows improved mechanical properties. The flexural and compressive strengths of concrete can be improved by the inclusion of only 1% natural fibers. These fibers can be used after special chemical treatment so that they will not decay quickly. The potential application of concrete reinforced with natural fibers can be in wall panels, interiors, roof tiles, etc;
5. Synthetic fibers, e.g., polyester, polypropylene, polyamide (nylon), and high-density polyethylene (HDPE), can be used to partially replace some part of cement, fine aggregates, or coarse aggregates in concrete. These fibers can enable concrete to avoid plastic shrinkage and increase resistance to cracking. The mechanical performance of the concrete can be improved only when the fiber percentage is between 1 and 2%. The possible applications of synthetic fiber-reinforced concrete can be in industrial floors, tunnels, canals, tiles, and residential construction projects;
6. Waste materials, such as biodegradable and non-biodegradable fibrous materials, can replace some part of cement and coarse aggregates. Use of fibrous waste materials in concrete improves some mechanical and chemical properties, and in addition, the waste material handling is carried out in an efficient way. Waste fibers from coconut, sugarcane, flax, hemp, bamboo, and kenaf used in concrete increase the ductility and the compressive strength when used at 0.5–2% content;
7. Some types of agrowaste (biomass), e.g., rice husk or wheat straw, can help in reducing the overall weight and density of the construction; however, they do not improve the mechanical performance significantly.

## 6. Future Strategies

In view of the potential of various waste fibers available in nature as well as from industry, a strategy for sustainable construction materials can be prepared for the future. This approach can significantly reduce the carbon emissions and burden of landfills. In addition, the mechanical performance of the construction materials can be improved with the inclusion of these fillers instead of small and large aggregates. A rigorous optimization technique can be employed to accurately determine the combination of materials and their contribution to individual performance attributes. A large data set can be created to enable an expert system and artificial intelligence/machine learning approach toward solving this complex yet essential problem for future constructions using eco-friendly and energy-efficient approaches.

## Figures and Tables

**Table 1:** Mechanical properties of selected high elastic modulus (HEM) fibers [11,12,13,14,15,16,17].

Sr. No.	Fiber	Density (Kg/m^3^)	Tensile Strength (MPa)	Elastic Modulus (GPa)	Elongation at Break (%)
1	Glass	2100	1000–3500	70–80	2.5–3.5
2	Basalt	1560	2600–4800	79.3–93.1	2.0–3.2
3	Carbon	1750	3500–7000	200–600	0.3–2.4

**Table 2:** Selected mechanical properties of low elastic modulus (LEM) fibers [25,26,27,28,29,30,31,32,33,34].

Sr. No	Fiber	Specific Gravity (Kg/m^3^)	Tensile Strength (MPa)	Elastic Modulus (GPa)	Elongation at Break (%)
1	Jute	1020–1480	490–800	10–30	1.16–1.93
2	Sisal	1400–1500	380–725	9.0–22.0	2.0–14.0
3	Banana	700–1350	150–900	27–32	0.35–9.54
4	Polyester	1330–1405	500–800	2.5–10.0	13.5–14.3
5	Polypropylene	900–910	300–750	0.5–3.0	15.0–30.0
6	Nylon	1130–1150	40–90	1.3–4.2	15.0–50.0
7	High-Density Polyethylene (HDPE)	940–970	23–29.5	0.4–2.5	600–1350

**Table 3:** Mechanical properties of biodegradable fibrous waste [69,71,99,110,116,126,130,132].

Sr. No.	Fiber	Density (Kg/m^3^)	Tensile Strength (MPa)	Elastic Modulus (GPa)	Elongation at Break (%)
1	Sugarcane bagasse	120–520	60–200	18–26	1.1–3.5
2	Coconut	1200–1500	170–500	2–8	17–25
3	Rice husk	321–425	70–80	-	-
4	Wheat straw	225–450	80–90	-	-
5	Bamboo	600–900	150–800	10–50	4.1–9.5
6	Flax	1300–1600	800–1500	50–150	1.2–3.7
7	Hemp	1400–1500	350–800	20–70	1.0–4.2
8	Kenaf	970	400–700	14–60	1.6–5.7

## Data Availability

No new data were created or analyzed in this study. Data sharing is not applicable to this article.
